# High generation of reactive oxygen species from neutrophils in patients with severe COVID-19

**DOI:** 10.1038/s41598-022-13825-7

**Published:** 2022-06-21

**Authors:** Tonny Veenith, Helena Martin, Martin Le Breuilly, Tony Whitehouse, Fang Gao-Smith, Niharika Duggal, Janet M. Lord, Rubina Mian, David Sarphie, Paul Moss

**Affiliations:** 1 Institute of Inflammation and Ageing, University of Birmingham, Birmingham, UK; 2 Institute of Immunology and Immunotherapy, University of Birmingham, Birmingham, UK; 3 University Hospitals Birmingham NHS Foundation Trust, Birmingham, UK; 4 Seroxo Limited, London, UK; 5 Birmingham Acute Care Research, University of Birmingham, Birmingham, UK

**Keywords:** Innate immunity, Translational research

## Abstract

Neutrophilia and an elevated neutrophil:lymphocyte ratio are both characteristic features of severe COVID-19 infection. However, functional neutrophil responses have been poorly investigated in this setting. We utilised a novel PMA-based stimulation assay to determine neutrophil-derived reactive oxygen species (ROS) generation in patients with severe COVID-19 infection, non-COVID related sepsis and healthy study participants. ROS production was markedly elevated in COVID-19 patients with median values ninefold higher than in healthy controls and was particularly high in patients on mechanical ventilation. ROS generation correlated strongly with neutrophil count and elevated levels were also seen in patients with non-COVID related sepsis. Relative values, adjusted for neutrophil count, were high in both groups but extreme low or high values were seen in two patients who died shortly after testing, potentially indicating a predictive value for neutrophil function. Our results show that the high levels of neutrophils observed in patients with COVID-19 and sepsis exhibit functional capacity for ROS generation. This may contribute to the clinical features of acute disease and represents a potential novel target for therapeutic intervention.

## Introduction

With the SARS-CoV-2 pandemic having led to an estimated 18 million deaths to date, there is an urgent need to increase understanding of the pathogenesis of severe disease, develop novel approaches to monitor disease progression, and guide clinical management. The natural history of COVID-19 is variable within different individuals although factors such as age, co-morbidity and increased body mass index (BMI) have been identified as determinants of increased risk^1^. An intense inflammatory process is the central feature in severe COVID-19 and there is increasing interest in the role of the innate immune system and its modulation in this process^2^. Improved understanding of the underlying mechanism, and a simple test for monitoring its progression, could represent a step change in clinical management.

Neutrophils are the most abundant immune cells in blood and play a crucial role in immune defence against bacterial, fungal and viral infections^3,4^. In patients with severe COVID-19 the neutrophil count typically increases whilst the lymphocyte count falls^5^ leading to an increase in the neutrophil to lymphocyte ratio (NLR)^6^. Four meta-analyses have now confirmed this pattern in severe COVID-19 infection^7–10^, but the biological basis for the association between neutrophilia and severe COVID-19 is currently unclear. In addition, there is increasing concern that neutrophils may contribute to the tissue damage associated with severe disease and lung autopsies show neutrophilic infiltration in pulmonary capillaries^11–14^. Increased levels of circulating DNA derived from neutrophil extracellular traps (NETs), indicative of neutrophil activation, have also been demonstrated^15^.

Neutrophils mediate many functional responses including generation of reactive oxygen species (ROS), also known as the ‘oxidative burst’. ROS generation can activate granular proteases and induce the formation of neutrophil extracellular traps (NETs). Additionally, ROS can cross the membranes of bacterial pathogens and damage their nucleic acids, proteins, and cell membranes. ROS generation is therefore a powerful antimicrobial weapon and a major component of innate immune defence against pathogens^3,16^. ROS are generated via the nicotinamide adenine dinucleotide phosphate (NADPH) oxidase complex^3,17,18^ from the membrane complex and intracellular pools^19^. Furthermore, ROS production triggers a number of downstream activities^20^ including release of cytoplasmic granules, NET generation, and stimulation of pro-inflammatory cytokine production including tumour necrosis factor alpha (TNFα) and macrophage inflammatory protein 2 (MIP-2)^21–23^. Strong ROS production can also mediate tissue damage and the oxidative burst must therefore be tightly regulated^20^. Indeed, release of superoxide radicals and H_2_O_2_ leads to oxidative stress that is thought to contribute to the cytokine storm and blood clot formation in SARS-CoV-2 infection^11,24^.

While the neutrophil count is an accurate marker of cell number, it does not assess cellular function. Indeed, an objective marker of neutrophil function could represent an important tool to assess pathogenesis and potentially guide patient management. We exploited a functional assay that has been developed to assess the capacity of leukocytes to produce ROS in response to phorbol 12-myristate 13-acetate (PMA) and luminol. This Leukocyte Coping Capacity (LCC) test (also known as the Leukocyte ImmunoTest, (LIT), Seroxo Ltd, UK) specifically and rapidly (10 min) quantifies leukocyte ROS release using a small volume of blood obtainable from capillary, arterial or venous sources. As such the LIT score provides a physiologically relevant means for monitoring the cellular capacity of leukocytes to produce superoxide radicals in real time. LIT also avoids centrifugation and ROS production can be studied under near physiological conditions.

Currently little is known regarding the production of ROS in patients with severe COVID. We compared the profile of ROS production from the blood of patients with severe COVID, non-covid related sepsis and healthy volunteers.

The specific objectives were to assess the performance of the LIT assay in patients with COVID and sepsis and to relate the values to neutrophil count and clinical outcome. We show that ROS levels are elevated in patients with acute sepsis and correlate strongly with the neutrophil count. The relative production of ROS after adjustment for neutrophil count was high in both groups but outlying values were seen in two patients with short term mortality.

## Methods

### Population

Ethical approval for this NIHR Urgent Public Health (UPH) Coronavirus Immunological Analysis (CIA) study was obtained from North West Preston Research Ethics Committee with favourable outcome (REC 20\NW\0240; Project ID: 282164) and conducted according to the Declaration of Helsinki and good clinical practice at the Intensive Care Unit (ICU) at the Queen Elizabeth Hospital, Birmingham. This work uses data provided by patients and collected by the NHS as part of their care and support at University Hospitals Birmingham NHS Foundation Trust. This data was processed under the extended control of patient information (COPI) notice (tinyurl.com/wwtbhzbj) in place during the study. The patient population consisted of patients who had previously been admitted to the ICU; the healthy volunteer population consisted of healthcare workers from the hospital. All research was performed in accordance with relevant guidelines/regulations. Analysis was performed on waste clinical samples or via finger-prick assay from healthy volunteers. ROS production in response to PMA was analysed within 30 min of collection.

Diagnosis of COVID status was confirmed using PCR. One patient had both COVID and sepsis. Sepsis patients were diagnosed using quick sequential organ failure assessment score (qSOFA) and all fulfilled the sepsis 3 criteria. All sepsis patients were defined with sepsis diagnosis by reference to qSOFA scoring (respiratory rate ≥ 22/min, altered mentation and systolic blood pressure ≤ 100 mm Hg). All patients with sepsis were hypotensive requiring vasopressors and had serum lactate greater than 2 mmol/L. No confounding factors were identifiable and bias was minimised by inclusive selection of a prospective cohort of patients. As this was an exploratory study with no known ‘effect size’ of response, formal power calculations were not performed at study onset.

### Data collection

The collected data included severity of COVID (according to WHO classification), age, sex, treatment, use of ventilation and type, lactate, co-morbidities including diabetes, PaO_2_/FiO_2_ ratio, use of antibiotics, days in ICU, neutrophil count, total leukocyte count.

### Measurement of ROS production by luminometer

ROS production was measured according to the method previously described^25–27^. Briefly, 10 μl samples of freshly obtained blood (obtained by finger prick or venepuncture) was added to 100 µl phosphate buffered saline (PBS) containing phorbol 12-myristate 13-acetate (PMA; Sigma) and luminol. The solution was incubated for 10 min at 37.5 °C. Chemiluminescence was quantified after 10 min using 3 M handheld luminometer (Clean-Trace, NG3) in relative light units (RLU). Triplicate LIT scores for neutrophil functionality level for each patient in these categories were averaged (mean LIT score) and these averages plotted.

### Statistics

Linear mixed-effects models where replicates are nested within subjects were estimated for both COVID-healthy subject samples and sepsis-healthy subject samples. These models enable the assessment of the degree of dependency among observations for the same subject. Given that the estimated Intraclass-correlation coefficient (ICC) in both models is extremely high (COVID-healthy: 0.9 and sepsis-healthy: 0.97), meaning that almost all of the variability in the data is due to between-subject variability, subsequent descriptive analysis and exploratory hypothesis testing has been performed on the means or medians of the three replicate LIT values for each subject.

For nominal variables, boxplots (or violin plots) show the distribution of scores by subgroup, and an independent t-test is used to test differences in LIT means between two independent groups. A Mann–Whitney U test is used when the assumptions of the independent t-test are not met. For ordinal or numerical variables, Spearman correlation coefficients have been computed, including confidence intervals and a test against the null hypothesis that the correlation is zero/the samples come from the same population. The 95% CI have been computed using a bootstrap method (number of replicates = 1000) which has to be interpreted with some caution in small studies.

## Results

**Figure 1:**
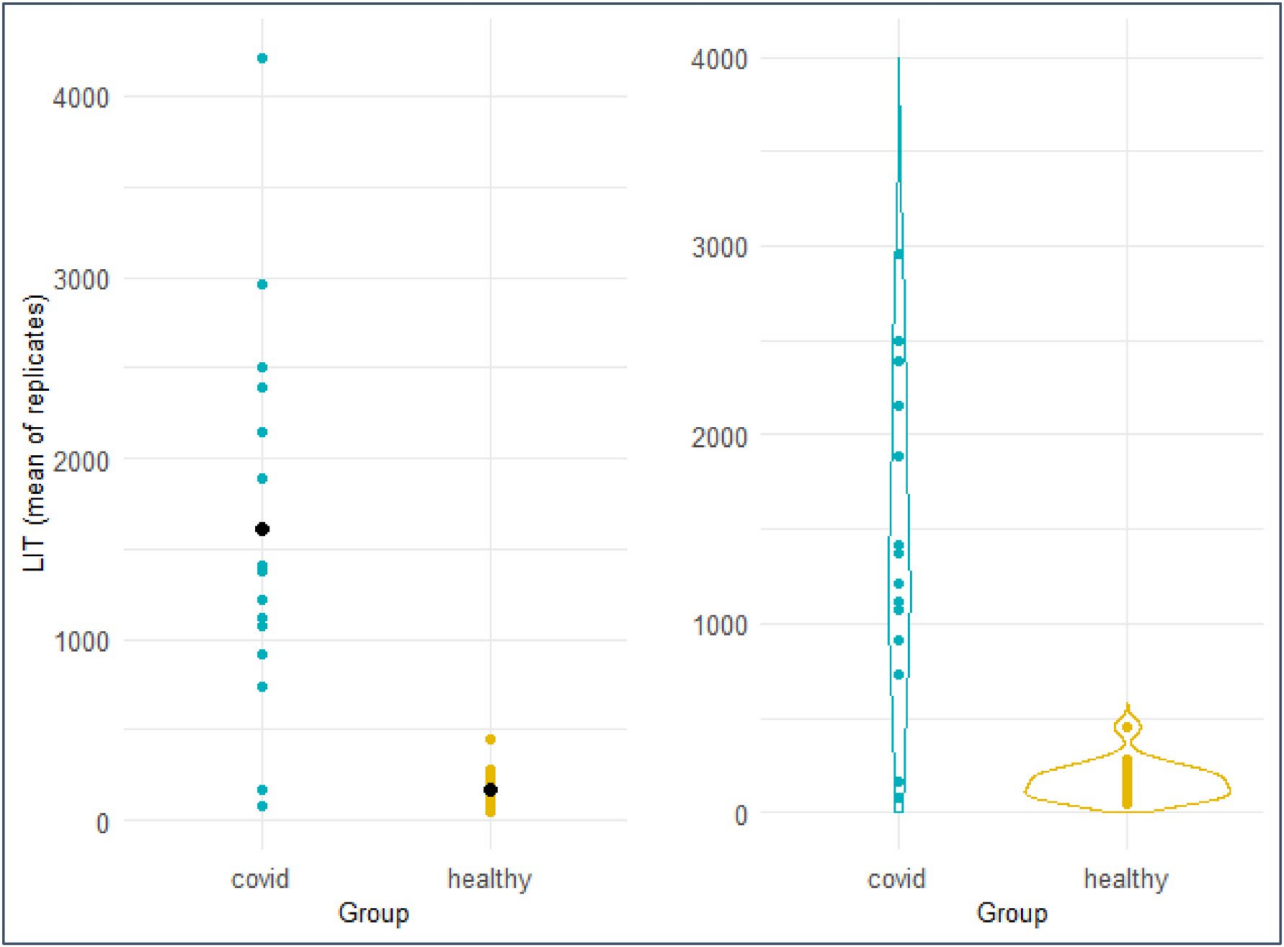
Dot plot (L) and violin plot (R) of LIT scores for patients with COVID-19 or healthy donors. Median LIT score for patients with COVID-19 was 1378 (IQR: 1276) and was ninefold higher than values within the control group (Median 153; IQR: 112, (Mann–Whitney U test, p < 0.05).

Patients with a confirmed COVID-19 positive PCR diagnosis and who were being treated within an ICU (n = 15) were enrolled into the study. ICU patients with non-COVID sepsis (n = 12) and healthy volunteers (n = 18) were also studied. Reactive oxygen species (ROS) production was measured by chemiluminescence detection on fresh blood samples and defined as a LIT™ score. Median LIT™ score for patients with COVID-19 was 1378 (IQR: 1276) and was ninefold higher than values within the control group (Median 153; IQR: 112, (Mann–Whitney U test, p <<< 0.05.) (Fig. 1).

**Figure 2:**
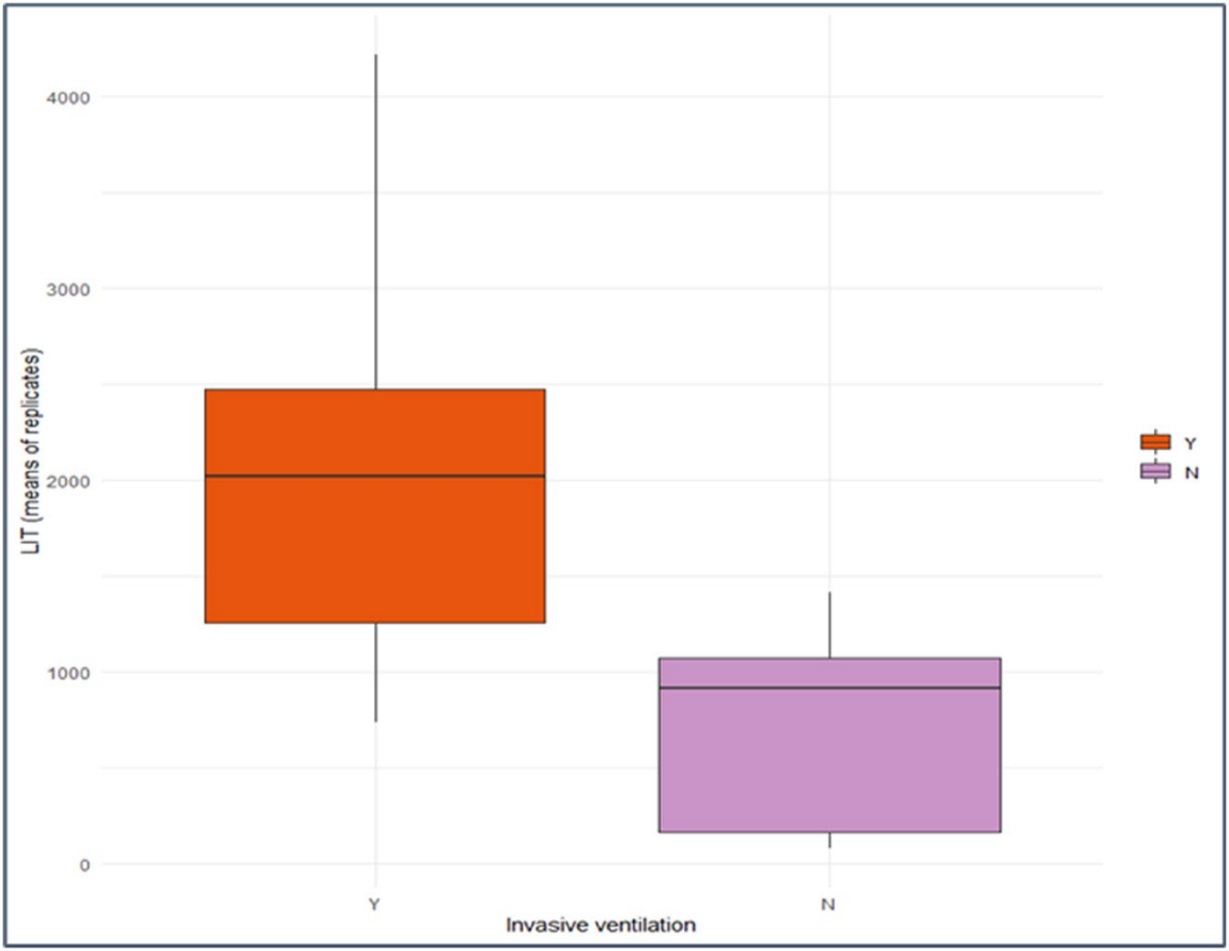
Box plots showing distribution of LIT scores in patients with COVID-19 in relation to invasive ventilation status. Horizontal lines in each box represent the median LIT scores for each cohort. Median LIT score for patients on invasive ventilation was 2020 and compared with 916 for those not on invasive ventilation (Mann Whitney U test: p = 0.02).

10 patients with COVID-19 were undergoing mechanical ventilation at the time of sampling and median LIT values were over twofold elevated in this group compared to non-ventilated patients (2020 vs 916 respectively; Mann Whitney U test: p = 0.02) (Fig. 2).

### ROS production is strongly correlated with neutrophil cell count

**Figure 3:**
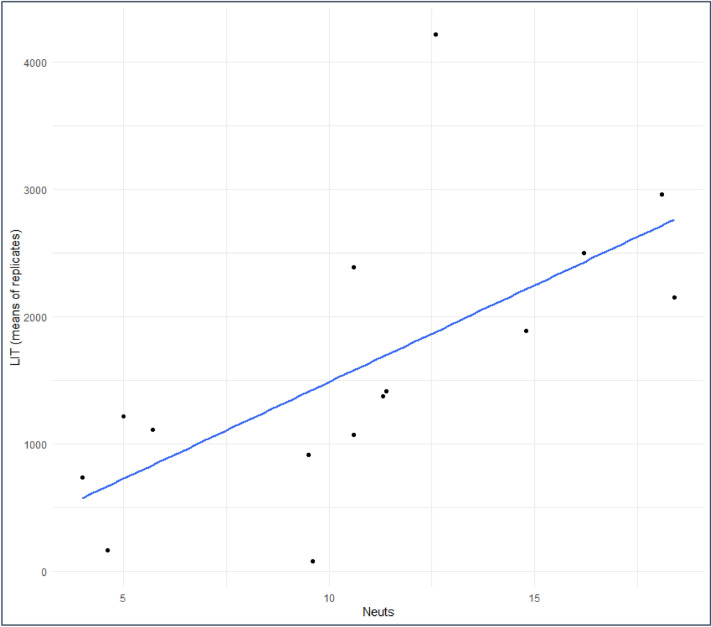
Scatterplot to show correlation between LIT and neutrophil count in patients with COVID-19. LIT scores and neutrophil counts are positively correlated (Spearman’s rho = 0.80 [0.52, 0.92] (p = 0.0004)).

We next went on to compare LIT values with haematological parameters such as total white blood cell count (WBC) and neutrophil count within the patient group. LIT values were found to correlate strongly with both values. Correlation with WBC was 0.73 (Spearman’s rho [0.39, 0.90]; (p = 0.003) with a higher value of 0.80 for the neutrophil count (Spearman’s rho [0.52, 0.92] (p = 0.0004)) (Fig. 3). These results support the assessment of the LIT score as a measure of the functional capacity of neutrophils within the blood.

### ROS production is also strongly correlated with neutrophil cell count in patients with non-COVID sepsis

In order to assess how the profile of ROS production in patients with COVID-19 compared to that seen in non-COVID-19 illness we assessed the samples from 12 ICU patients with non-COVID-19 related sepsis.

**Figure 4:**
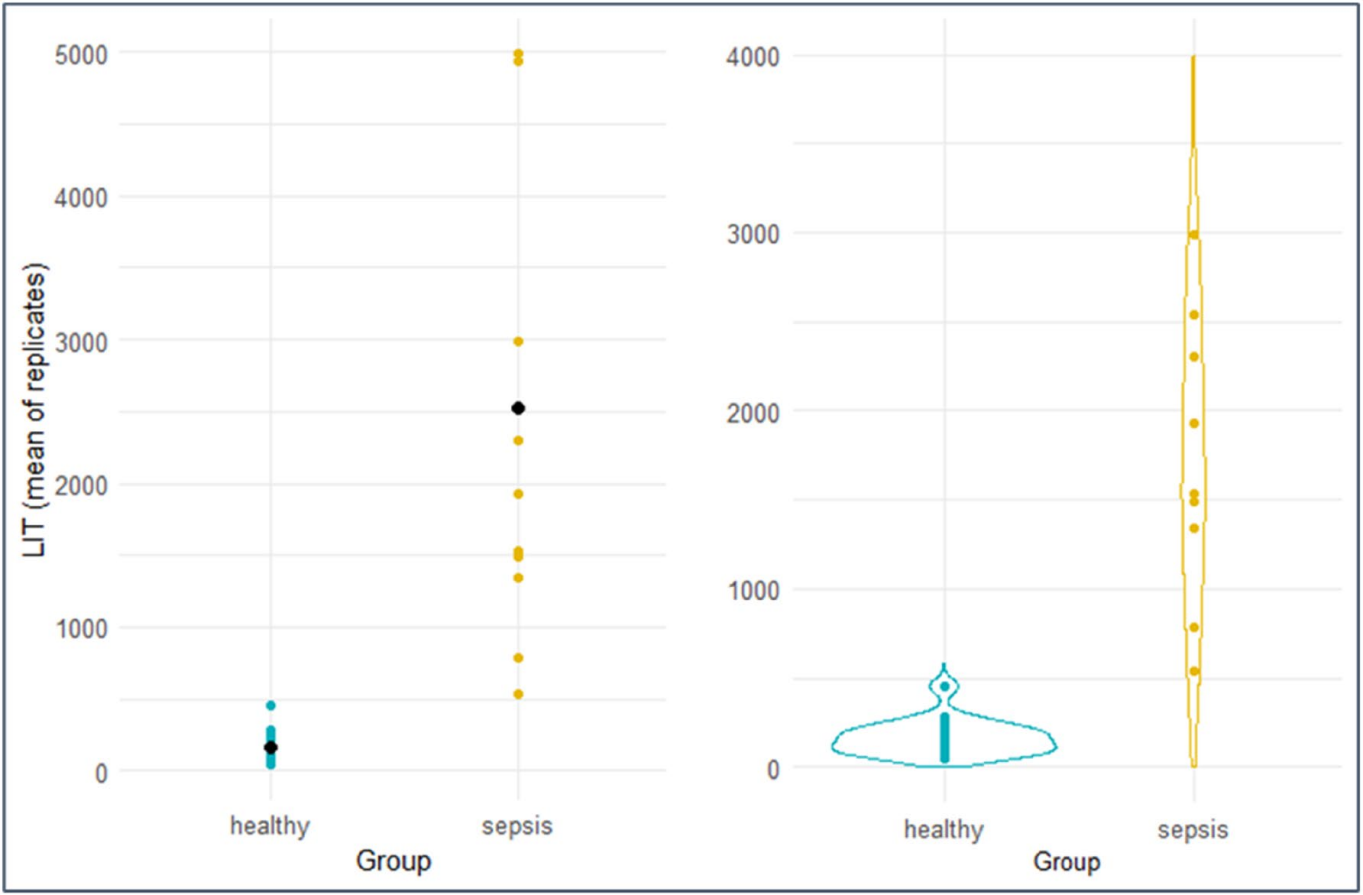
Dot plot (L) and violin plot (R) of LIT scores in patients with sepsis compared to healthy volunteers. Median LIT score for patients with sepsis was 2120 (IQR: 2024) and was > 13-fold higher than values within the control group (median: 153; IQR: 112, Mann–Whitney U test, p < 0.05).

The median LIT™ score within this patient cohort was 2120 (IQR: 2024), which was 14-fold higher than the median value of 153 (IQR:112) in volunteer cohort, demonstrating that peripheral ROS production is also increased in patients with severe non-COVID infection (Fig. 4) (Mann–Whitney U test, p < 0.05). No association was found between LIT™ score and the use of invasive ventilation within this group (Mann–Whitney U test: p-value = 0.88). As with COVID-19 patients, LIT™ scores correlated strongly with the peripheral neutrophil count (R = 0.76 [0.18, 1.00] (p = 0.009). As such these results demonstrate high levels of ROS production from activated blood samples from ICU patients with sepsis and suggest a potential utility as a tool to monitor sepsis progression and management.

### The ratio of ROS production normalised to neutrophil count may act as a potential guide to risk of short term mortality

Given that the LIT score correlated broadly with the neutrophil count we next went on to investigate this association in more detail between the cohorts. In particular, the total LIT score was divided by the peripheral neutrophil count to assess ‘ROS production per cell’ and termed as the LIT-N value (Table 1). The potential importance of LIT-N in relation to clinical outcome was next assessed in relation to the 28-day mortality of patients. The healthy controls did not have a blood count performed and as such it was not possible to determine the LIT-N value in this cohort.

**Table 1:** Clinical characteristics of patient cohort.

Age (years)	Diagnosis	Day of disease	SOFA score	Sex	Diagnosis	CXR	WBC × 10^9/L	Neuts × 10^9/L	Alive at 28 days	Invasive ventilation	LIT Score	LIT-N
20–29	Sepsis	3	18	M	Ventilator-associated pneumonia	Lobar pneumonia	2.4	1.4	Y	Y	787	562
40–49	Sepsis	11	16	M	Ventilator-associated pneumonia	Lobar pneumonia	13	11.9	N	Y	4983	419
40–49	Sepsis	6	18	M	Ventilator-associated pneumonia	Lobar pneumonia	5.1	3.1	Y	Y	540	174
60–69	Sepsis	3	12	F	Ventilator-associated pneumonia	Lobar pneumonia	10.6	9	Y	N	1340	149
70–79	Sepsis	3	12	F	Ventilator-associated pneumonia	Lobar pneumonia	20.6	18.9	Y	N	2305	122
50–59	Sepsis	14	12	M	Meningitis and ventriculitis	N/A	10.9	8.9	N	Y	1535	172
70–79	Sepsis	6	16	M	Community acquired pneumonia	Lobar pneumonia	19.1	15.2	N	Y	2986	196
40–49	Sepsis	22	11	M	Ventilator-associated pneumonia	Lobar pneumonia	6.9	5.8	Y	Y	1487	256
50–59	Sepsis	9	10	M	Intra-abdominal sepsis	N/A	42.2	40.8	Y	Y	4942	121
30–39	Sepsis	5	12	F	Intra-abdominal sepsis	N/A	18.3	15.4	Y	N	2538	165
70–79	Sepsis	1	12	F	Intra-abdominal sepsis	N/A	14.9	12.9	Y	N	1934	150
50–59	Sepsis	1	12	F	Intra-abdominal sepsis	N/A	24.3	22.9	Y	N	4938	216
											Average	225
50–59	COVID	9	13	M	COVID, no bacterial infection	Four quadrant infiltrate	18.7	16.2	Y	Y	2501	154
50–59	COVID	1	10	M	COVID, no bacterial infection	Four quadrant infiltrate	5.7	5	Y	Y	1217	243
60–69	COVID	10	13	M	COVID, concurrent bacterial infection	Four quadrant infiltrate	16.3	10.6	Y	Y	2390	225
40–49	COVID	11	14	F	COVID, no bacterial infection	Four quadrant infiltrate	21.7	18.1	Y	Y	2959	163
60–69	COVID	1	12	M	COVID, no bacterial infection	Four quadrant infiltrate	11.5	10.6	Y	N	1073	101
60–69	COVID	6	13	M	COVID, no bacterial infection	Four quadrant infiltrate	13.7	12.6	Y	Y	4217	335
50–59	COVID	20	16	M	COVID, no bacterial infection	Four quadrant infiltrate	4.9	4	Y	Y	935	234
40–49	COVID	2	14	M	COVID, no bacterial infection	Four quadrant infiltrate	13.1	11.4	Y	N	1414	124
50–59	COVID	14	14	F	COVID, no bacterial infection	Four quadrant infiltrate	7.9	5.7	Y	Y	1115	196
50–59	COVID	2	12	M	COVID, no bacterial infection	Four quadrant infiltrate	5.8	4.6	Y	N	169	37
50–59	COVID	6	11	M	COVID, no bacterial infection	Four quadrant infiltrate	10.5	9.6	N	N	84	8.75
50–59	COVID	15	12	M	COVID, concurrent bacterial infection	Four quadrant infiltrate	14.8	9.5	Y	N	916	96
50–59	COVID	19	14	M	COVID, concurrent bacterial infection	Four quadrant infiltrate	18	14.8	Y	Y	1891	128
50–59	COVID	32	12	M	COVID, concurrent bacterial infection	Four quadrant infiltrate	15.4	11.3	Y	Y	1679	149
50–59	COVID	30	16	M	COVID, concurrent bacterial infection	Four quadrant infiltrate	22.2	18.4	Y	Y	2150	117
											Average	154

The average LIT-N score in patients with COVID-19 or sepsis was 154 and 225 respectively. 4 out of the 26 patients with an available neutrophil count died within the study period of which 2 succumbed within 24 h of the LIT™ test. One of these two patients had COVID-19 infection and here the personal LIT-N value was markedly low at 9. The second patient had non-COVID sepsis and here the LIT-N score was the highest level in the cohort at 419 prior to death. The other two patients had LIT-N scores closer to the mean of their group (LIT-N = 172 and LIT-N = 196) but death did not occur until 4 and 26 days respectively after testing.

These observations require further assessment on a larger cohort but may indicate that patients with extreme hypo- or hyperfunctional neutrophil activity may be at markedly increased risk of short term mortality. As such the LIT-N score could be of potential value in clinical management.

## Discussion

Neutrophils are critical mediators of the innate immune response to acute infection and inflammation^28,29^. However, their strong functional capacity may contribute to tissue damage in patients with severe illness. Currently, clinical assessment of neutrophils is performed by measurement of the total cell count. In this paper we provide proof of concept data to enhance this analysis through measurement of the functional capacity of neutrophils to generate reactive oxygen species. Our findings indicate a range of novel observations regarding neutrophil function in COVID-19 and sepsis.

The first striking finding was the substantially increased total ROS values in patients with severe COVID-19 infection and sepsis. The median values in COVID-19 were ninefold higher than in healthy donors and consistent with previous reports of neutrophilia and high levels of ROS production in this patient group. The assessment of patients with sepsis due to causes other than COVID-19 also revealed high levels of ROS production, revealing that increased values may be a consistent feature of patients with acute and severe infection. This is noteworthy as ROS production can activate a range of downstream inflammatory processes that may contribute to tissue damage^21–23,30^.

As expected, LIT values were strongly correlated with the peripheral neutrophil count. However, we were particularly interested to assess the relative profile of ROS production in relation to the neutrophil count and therefore developed the LIT-N score as a measurement of the increment in LIT in relation to a 1-unit increase in total neutrophil count. LIT-N values were 45% higher in patients with sepsis compared to COVID-19 infection and may relate to the immature or dysfunctional mature neutrophil phenotype that has been reported in severe COVID-19 patients. In addition, viral infection may potentially differentially regulate ROS production from the plasma or granular membranes^19^. Neutrophils do express ACE2, the receptor for the virus spike protein, and SARS-CoV-2 can directly stimulate NETosis and intracellular ROS production within neutrophils although there is currently no clear evidence for intracellular infection^31^.

It is possible that high ROS production from neutrophilia contributes to the characteristic profile of tissue damage in patients with severe COVID-19 infection. Indeed, neutrophil infiltration has been suggested to contribute to pulmonary complications^32,33^, and ROS release contributes both to cytokine storm and blood clot formation in SARS-CoV-2 infection^11,24^. Patients who were undergoing mechanical ventilation produced significantly higher levels of ROS although it is not clear if this is a result of the forced ventilation or simply a reflection of the disease progression.

Bacterial infection has the potential to modulate ROS production and, as concurrent bacterial infection is a common feature of severe COVID-19, it was of interest to assess the influence of co-infection on LIT-N score. LIT-N scores within the COVID-19 cohort were 160 for patients with concurrent bacterial infection compared to 143 for those without. As such, no significant interaction was observed but this will be an important area for future study.

We are particularly interested to determine whether the LIT and LIT-N scores can be of value in helping to predict outcome and guide treatment management during acute infection. Assessment of short-term mortality showed that two patients who died had LIT-N scores that were markedly departing from the respective means for the patient groups. In particular, the patient who succumbed to COVID-19 had extremely low levels of LIT-N, indicating that neutrophils were hypofunctional prior to death. As such, this suggests that neutrophil exhaustion or a higher frequency of immature neutrophils^34^ may be a marker of severe outcome and this deserves further assessment in larger cohorts. In contrast, the patient with non-COVID sepsis showed a markedly elevated LIT-N score and may indicate that extreme hyperactivation of neutrophil function can also be prognostic of poor short-term outcome. To what extent these differential levels of ROS may themselves contribute to the development of such severe disease is also worthy of further consideration.

The limitations of our study include lack of neutrophil count within the healthy control donors and relatively low level of mortality in the disease groups with which to assess prognostic implications of the LIT-N score. Ongoing larger studies are in progress to address these issues.

In summary, our results indicate that the LIT™ test and a derived indicator of LIT-values in relation to neutrophil count (LIT-N) provides a potentially powerful new tool to assess the functional capacity of neutrophils in patients with severe infection. Current laboratory assays of immune cell function rely almost exclusively on measurement of cell number but do not consider their functional capacity^35^. Our findings pave the way for a new assessment of cellular function as a rapid point-of-care test that may help to monitor disease progression. Furthermore, this may also hold potential for guiding the introduction of immunomodulatory therapies to optimize innate cell function and improve clinical outcome.

## Data Availability

Supporting data and material is available upon request.
